# Effect of Probiotics Supplementation on REM Sleep Behavior Disorder and Motor Symptoms in Parkinson's Disease: A Pilot Study

**DOI:** 10.1111/cns.70541

**Published:** 2025-07-27

**Authors:** Yitong Du, Lin Wang, Ying Cui, Xiaojiao Xu, Mingkai Zhang, Yue Li, Ting Gao, Dan Gao, Zhi Sheng, Shiya Wang, Houzhen Tuo

**Affiliations:** ^1^ Department of Neurology Beijing Friendship Hospital, Capital Medical University Beijing China; ^2^ Department of Neurology The First Medical Centre, Chinese PLA General Hospital Beijing China; ^3^ Nanfang Hospital Baiyun Branch Guangdong China; ^4^ Editorial Department Chinese Medical Journal, Chinese Medical Association Publishing House Beijing China; ^5^ Beijing Renhe Hospital Beijing China; ^6^ Shijingshan Teaching Hospital of Capital Medical University, Beijing Shijingshan Hospital Beijing China

**Keywords:** gut microbiota, motor symptoms, Parkinson's disease, probiotics, REM sleep behavior disorder

## Abstract

**Background:**

Parkinson's disease (PD) patients experience gut microbiota dysbiosis. Probiotic intervention could potentially serve as a safe and effective adjunctive therapeutic approach for PD, but its effects on rapid eye movement sleep behavior disorder (RBD) and motor symptoms in PD patients warrant further investigation.

**Objectives:**

To examine the influence of probiotics on RBD, motor symptoms, gut microbiota, and serum metabolites in individuals with PD.

**Methods:**

In this randomized controlled trial, PD patients were randomly allocated to either a probiotics or a control group while maintaining standard treatments. Clinical outcomes, including Unified Parkinson's Disease Rating Scale (UPDRS) and RBD Questionnaire‐Hong Kong (RBDQ‐HK) were assessed at baseline and post‐treatment. Furthermore, fecal and blood samples were collected from PD patients at both timepoints, with additional samples obtained from healthy controls for comparison. The 16S rRNA gene V3‐V4 region sequencing method was used to analyze gut microbiota composition, and untargeted metabolomic techniques were utilized to assess serum metabolite alterations, followed by correlation analysis.

**Results:**

Fifty eligible PD patients were enrolled and randomly allocated into two groups. After 12 weeks of intervention, the probiotic group showed significant reductions in both UPDRS total scores (−4.8 ± 7.5 vs. 1.8 ± 13.1, *p* = 0.009) and RBDQ‐HK scores (−7.5 ± 6.5 vs. 0 ± 5.8, *p* = 0.015) compared to controls. Gut microbiota analysis revealed increased abundance of *Actinobacteria*, *Negativicutes*, and *Bacillus*, with reductions in *Lactococcus*, *Comamonas*, and *Enterococcus* after probiotic intervention. Furthermore, compared to normal controls, PD patients exhibited 9 significantly elevated and 11 significantly reduced metabolites; probiotic intervention altered the serum metabolome in PD patients.

**Conclusions:**

This study demonstrated probiotics' potential to ameliorate RBD and motor symptoms while positively affecting the composition of the gut microbiota and serum metabolites in PD patients.

## Graphical abstract

Twelve‐week probiotics supplementation in Parkinson's disease (PD) patients demonstrated significant improvements in motor symptoms and REM sleep behavior disorder (RBD) symptoms. Additionally, the intervention exerted a modulatory effect on gut microbiota composition and plasma metabolite profiles, suggesting a potential role of probiotics in modulating the gut‐brain axis in PD.

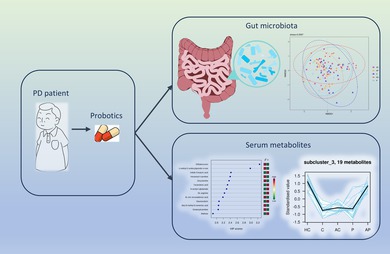

## 1 Introduction

Parkinson's disease (PD) is a neurodegenerative disorder characterized by a high disability rate, with clinical symptoms encompassing both motor and non‐motor symptoms [1]. Current treatment for PD, such as levodopa, dopamine agonists, and monoamine oxidase B inhibitors, can only partially control motor symptoms but does not prevent dopaminergic neuron degeneration [2]. As the disease progresses, patients may develop motor complications such as the “on–off” phenomenon, end‐of‐dose wearing‐off, and dyskinesia that increase the burden on patients' families and society.

Rapid eye movement sleep behavior disorder (RBD) is present in 40%–69% of PD and is considered one of the most promising prodromal PD markers. RBD is characterized by dream‐related complex motor behaviors and loss of normal skeletal muscle atonia during REM sleep [3, 4]. Current PD medications fail to improve RBD symptoms, and some drugs even exacerbate the frequency of RBD episodes frequency [5]. Although clonazepam is recommended for the management of RBD, its long‐term use may lead to side effects, including cognitive decline.

In recent years, studies have highlighted the microbiota‐gut‐brain axis's crucial role in PD pathogenesis and have put forth the intestinal origin hypothesis [6, 7, 8]. Some evidence suggests gut dysbiosis in PD patients; aberrant aggregation and impaired clearance of α‐synuclein in the enteric nervous system (ENS) may be associated with intestinal flora [9, 10, 11]. Consequently, modulating gut microbiota composition has emerged as a novel therapeutic strategy [12]. Probiotics can maintain intestinal epithelial integrity, protect the intestinal barrier, regulate gastrointestinal immunity, and inhibit pathogenic bacteria [13]. Recent research has demonstrated that probiotics not only contribute significantly to the prevention and treatment of various gastrointestinal diseases [14], but also benefit conditions like diabetes, malignancies, cardiovascular disorders, and central nervous system disease, including Alzheimer's disease (AD), depression, PD, epilepsy, and other diseases [15, 16, 17, 18]. This investigation aimed to evaluate the impact of probiotics supplementation on motor symptoms and RBD in PD patients, and to investigate probiotics‐induced changes in gut microbiota and serum metabolites using 16S rRNA gene sequencing and metabolomics analysis.

## 2 Methods

### 2.1 Study Design and Participants

This 12‐week, open‐label, randomized controlled trial was approved by the Ethics Review Committee of Beijing Friendship Hospital, Capital Medical University (No. 2018‐P2‐080‐01). All participants provided informed consent, and all measurements and questionnaires were voluntary. The trial was registered in the Chinese Clinical Trial Registry (ChiCTR2200065638).

We recruited idiopathic PD patients from December 2017 to December 2020. Inclusion criteria: (1) Idiopathic PD patients, meeting the 2015 Movement Disorder Society (MDS) clinical diagnostic criteria; (2) Aged 40–80 years; (3) Modified Hoehn and Yahr stages 1–3, with stable motor symptoms for ≥ 1 month before enrollment; (4) No changes to PD medications in the 4 weeks preceding enrollment; (5) Understand and agree with this clinical trial. Exclusion criteria were: (1) Use of probiotics, prebiotics, or antibiotics within 8 weeks before enrollment; (2) History of gastrointestinal tumors, inflammatory bowel disease, or gastrointestinal surgery; (3) Severe cardiovascular, hepatic, or renal dysfunction, or otherwise unable to cooperate with the study; (4) Known allergy to the study probiotics.

Thirty sex‐ and age‐matched healthy controls were recruited. Inclusion criteria: Aged 40–80 years; no neurological disorders. Exclusion criteria matched those of PD subjects.

### 2.2 Intervention

All participants were randomly assigned to either the probiotics group or the control group. Both groups maintained their original treatment regimens unchanged, while the probiotics group additionally received 2 capsules of *Bacillus licheniformis* three times a day and 4 capsules of BIFICO twice a day for 12 weeks. Participants were instructed to maintain their habitual diets throughout the study. Each patient was followed up face‐to‐face every month to assess protocol adherence and document adverse events.

### 2.3 Probiotics Formulation

The probiotics mixture used in this study contained four bacterial species, *Bacillus licheniformis* (2.5 × 10^9^ CFU/capsule, Dongbei Pharmaceutical Group Company, Shenyang No. 1 Pharmaceutical Co. Ltd., China), *Bifidobacterium longum* , *Lactobacillus acidophilus* , and *Enterococcus faecalis* (each 1.0 × 10^7^ CFU/capsule, Shanghai Sine Pharmaceutical Co. Ltd., China).

### 2.4 Assessment of Outcomes

The primary outcome was the Unified Parkinson's Disease Rating Scale (UPDRS) total score. The secondary outcome measurements included REM sleep behavior disorder questionnaire‐Hong Kong (RBDQ‐HK) scores, levodopa equivalent daily dosage (LEDD), alteration of gut microbiota, and serum metabolites.

### 2.5 Clinical Assessments

Demographic data, disease duration, and detailed medical history, including medications, were collected. Using Modified H&Y to assess the stage of the disease.

All outcome assessments were conducted at baseline and week 12. Using the RBD screening questionnaire (RBD‐SQ) for preliminary screening of RBD symptoms [19], patients whose RBD‐SQ score is ≥ 6 points will be further evaluated with the RBD‐HK scale [20].

### 2.6 Fecal Sample Collection

Participants were instructed to collect fecal samples at baseline and week 12 using fecal collection containers provided by Allwegene Company (Beijing). All samples were stored at‐80°C until processing.

### 2.7 Blood Sample Collection

For each subject, a 3 mL fasting blood sample was collected at both baseline and 12 weeks, with all blood draws conducted between 8:00 AM and 12:00 PM. The samples were anticoagulated with heparin and centrifuged at 1800 rpm for 15 min, then the separated plasma specimens were stored in a −80°C freezer.

### 2.8 DNA Extraction and 16S rRNA Gene Sequencing

Fecal genomic DNA was extracted according to previous descriptions [21]. The V3‐V4 hypervariable regions of bacterial 16S rRNA gene were amplified with universal primers 338F (5′‐ACTCCTACGGGAGGCAGCAG‐3′) and 806R (5′‐GGACTACHVGGGTWTCTAAT‐3′). Paired‐end sequencing was conducted on the Illumina MiSeq PE300 high‐throughput sequencing platform (Allwegene Company, Beijing, China).

### 2.9 Metabolomic Analysis

Serum metabolite extraction and analysis were assisted by the Beijing Allwegene Company. For each sample, 50 μL was aliquoted into an EP tube. Subsequently, 200 μL of extraction solution (acetonitrile: methanol = 1: 1, containing isotopically labeled internal standard mixture) was added. After vortexing for 30 s, samples were sonicated for 10 min in an ice‐water bath and incubated for 1 h at −40°C to precipitate proteins. Then the samples were centrifuged at 12 000 rpm for 15 min at 4°C. The resulting supernatant was transferred to a fresh glass vial for LC/MS analysis. The quality control sample was prepared by mixing equal aliquots of the supernatants from all of the samples. In this study, 71 samples were included for non‐targeted metabolomics analysis: Normal controls (15 samples), Control group (26 samples), and Probiotics group (30 samples).

### 2.10 Statistical Analysis

SPSS 21.0 (SPSS Inc., Chicago, IL, USA) was used for the statistical analysis. Normality of variables (age, Hoehn‐Yahr stage, UPDRS, UPDRS‐III, RBD‐HK, and LEDD) was assessed using Shapiro–Wilk tests. The results demonstrated that age and UPDRS scores in both groups followed normal distribution, whereas Hoehn‐Yahr stage, UPDRS‐III scores, and LEDD in the control group did not conform to the normal distribution. For comparison of normally and non‐normally distributed continuous data, paired t‐test (age, UPDRS) or Wilcoxon signed rank test (Hoehn‐Yahr stage, UPDRS‐III scores, and LEDD) were used for within‐group differences, t‐test (age, UPDRS) and Mann–Whitney U test (Hoehn‐Yahr stage, UPDRS‐III scores, and LEDD) were used respectively for between‐group differences. Categorical variables were expressed as a composition ratio, using the Chi‐square test for between‐group comparisons. Spearman's rank‐order correlation analysis was used to assess non‐parametric associations between variables. Two‐sided *P* values < 0.05 were considered to indicate statistical significance.

For bioinformatic analysis, raw sequencing data were demultiplexed using QIIME1 (v1.8.0) based on barcode sequences, and the data were filtered and spliced using PEAR (v0.9.6). Sequences shorter than 230 bp were removed, and the UCHIME method was used according to the Gold Database to remove the chimeric sequences. Qualified reads were clustered into operational taxonomic units (OTUs) at 97% similarity using the UPARSE algorithm of VSEARCH (v2.7.1) software [22]. The Ribosomal Database Project (RDP) Classifier tool was used to classify all sequences into different taxonomic groups against the SILVA128 database [23].

Then alpha and beta diversity indices were calculated using Quantitative Insights Into Microbial Ecology (QIIME) [24]. Principal coordinates analysis (PCoA) was performed in R (v3.6.0) software based on Weighted Unifrace distances [25]. The metastats intergroup heterogeneity analysis was performed using Mothur (v.1.34.4) software, and LEfSe analysis was performed using Python (v2.7) software (LDA score > 3.0 and *p* < 0.05) [26]. Microbiota functional potential was predicted using PICRUSt 2.

MetaboAnalyst 3.0 was used to analyze metabolomics data, including principal component analysis (PCA) and orthogonal partial least squares discrimination analysis (OPLS‐DA). Metabolites with Variable Importance in the Projection (VIP) > 1 and *p* < 0.05 (student's t test) were considered statistically significant. The KEGG database was used for metabolic pathway analysis of differential metabolites.

## 3 Results

**FIGURE 1:**
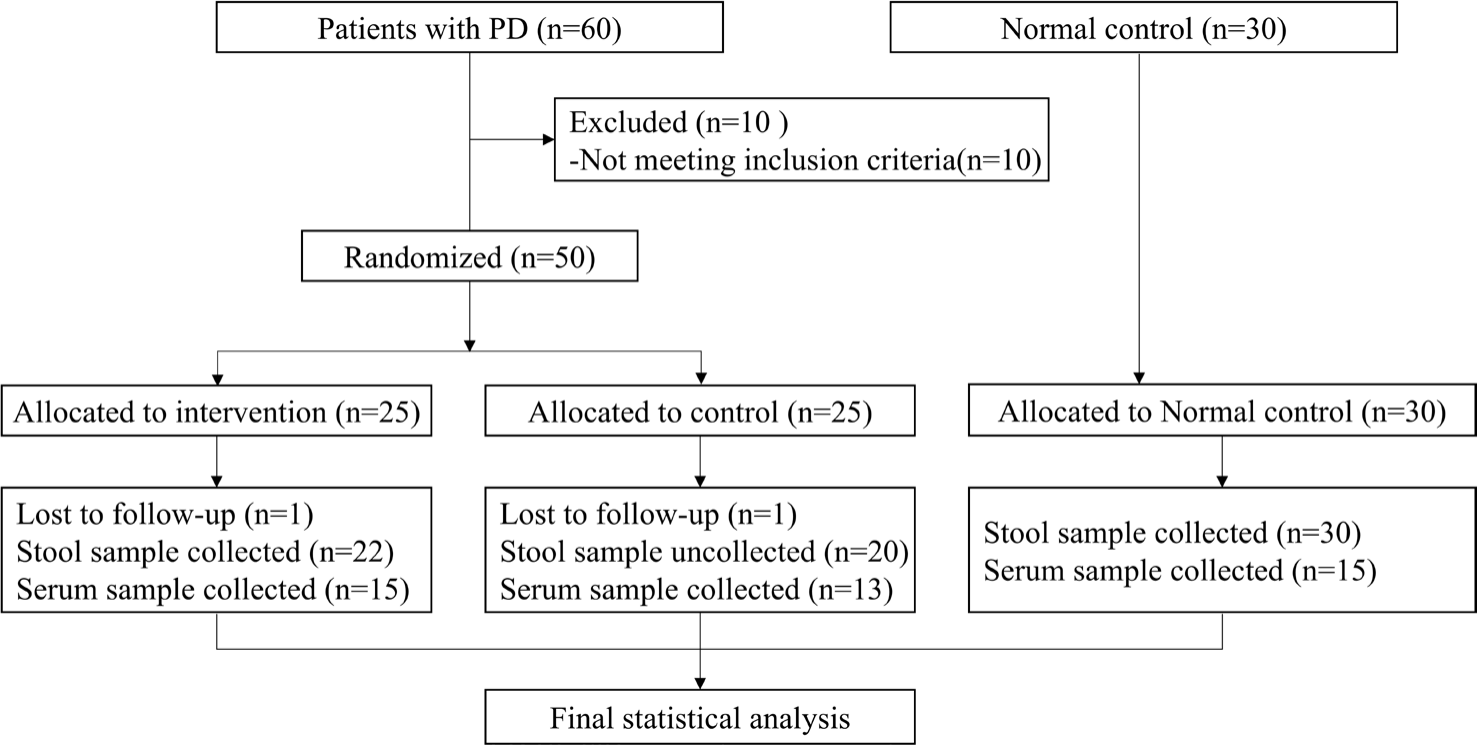
Study flow diagram.

We initially screened 60 patients, of whom 50 met the inclusion criteria without meeting any exclusion criteria. All 50 participants were randomly allocated to either the probiotics group (*n* = 25) or the control group (*n* = 25). Two patients (one per group) withdrew from the study for personal reasons (Figure 1). All 50 cases were ultimately included in the analysis by intention‐to‐treat (ITT) analysis. In addition, samples from normal controls were retained for subsequent studies.

General characteristics, PD severity, and medications showed no statistically significant differences between groups (Table 1).

**TABLE 1:** Baseline characteristics and intragroup versus intergroup comparisons of clinical parameters.

	Probiotics group (*n* = 25)				Control group (*n* = 25)				*p*‐value (between groups)
	Baseline	End‐of‐trail	Change	*p*‐value	Baseline	End‐of‐trail	Change	*p*‐value	
Age (years)	66.7 ± 9.1	—	—	—	64.8 ± 8.7	—	—	—	0.48^a^
Males, *n* (%)	72.0	—	—	—	48.0	—	—	—	0.08^b^
Modified H&Y	2 (2, 2.5)	—	—	—	2 (1.5, 2.5)	—	—	—	0.98^c^
Levodopa, *n* (%)	20 (80)	—	—	—	23 (92)	—	—	—	0.22^b^
UPDRS‐III	20.4 ± 7.8	—	—	—	17.7 ± 10.8	—		—	0.14^c^
UPDRS	31.2 ± 9.9	26.4 ± 11.2	−4.8 ± 7.5	0.004^d^	27.9 ± 14.7	29.7 ± 14.4	1.8 ± 13.1	0.34^d^	0.009^f^
LEDD (mg/day)	351.5 ± 194.6	330.0 ± 183.7	−21.5 ± 50.4	0.048^d^	325 (162.5, 425)	325 (187.5, 512.5)	0 (0, 50)	0.012^e^	0.004^f^
RBD‐HK	35.9 ± 11.9 (*n* = 21)	28.4 ± 12.2	−7.5 ± 6.5	< 0.001^d^	30.6 ± 11.3 (*n* = 17)	30.6 ± 11.1	0 ± 5.8	1^d^	0.015^f^
RBD improvement cases	0	8	—	—	0	1	—	—	0.02^b^

Abbreviations: LEDD, levodopa equivalent daily dosage; RBDQ‐HK, REM sleep behavior disorder questionnaire‐Hong Kong; UPDRS, Unified Parkinson's Disease Rating Scale; UPDRS‐III, Unified Parkinson's Disease Rating Scale‐III.

^a^ *p*‐values obtained from unpaired *t*‐test.

^b^ *p*‐values obtained from Chi‐square test.

^c^ *p*‐values obtained from Mann–Whitney U test.

^d^ *p*‐values obtained from paired *t*‐test.

^e^ *p*‐values obtained from Wilcoxon Signed rank test.

^f^ *p*‐values obtained from the time × group interaction.

### 3.1 Effect of Probiotics on RBD in PD Patients

The probiotics group included 21 cases with RBD, compared to 17 in the control group. At baseline, there was no statistically significant difference in RBD‐HK scores between the two groups. After 12 weeks of treatment, the probiotics group showed an average decrease of 7.5 points in the RBD‐HK score, whereas the control group showed no significant change (−7.5 ± 6.5 vs. 0.0 ± 5.8, *p* = 0.015, Table 1). A reduction of > 30% in RBD‐HK scores was considered symptomatic improvement. Eight patients in the probiotics group achieved this threshold, compared to only one in the control group. Consequently, the probiotics group showed a significantly higher improvement rate for RBD (38.1% vs. 5.9%, *X* ^2^ = 5.39, *p* = 0.02; Table 1). These results suggest that probiotics treatment could alleviate RBD symptoms in PD patients.

### 3.2 Effects of Probiotics on Motor Symptoms in PD Patients

Compared to baseline, UPDRS scores decreased significantly in the probiotics group but increased slightly in the control group (−5.2 ± 8.5 vs. 1.8 ± 13.1, *p* = 0.028). Additionally, the LEDD decreased in the probiotics group after probiotics supplementation(351.5 ± 194.6 vs. 330.0 ± 183.7, *p* = 0.048), with a greater reduction trend compared with the control group (Table 1). These results indicated that adjunctive probiotics therapy may enhance motor symptoms and reduce the levodopa equivalent dosage in PD patients compared to conventional treatment alone.

### 3.3 Effects of Probiotics on Gut Microbiota in PD Patients

Forty‐two participants (probiotics group: *n* = 22; control group: *n* = 20) provided fecal samples meeting analytical standards at both baseline and post‐treatment. All participants maintained stable dietary habits during the treatment. Additionally, 30 normal controls provided stool samples.

**FIGURE 2:**
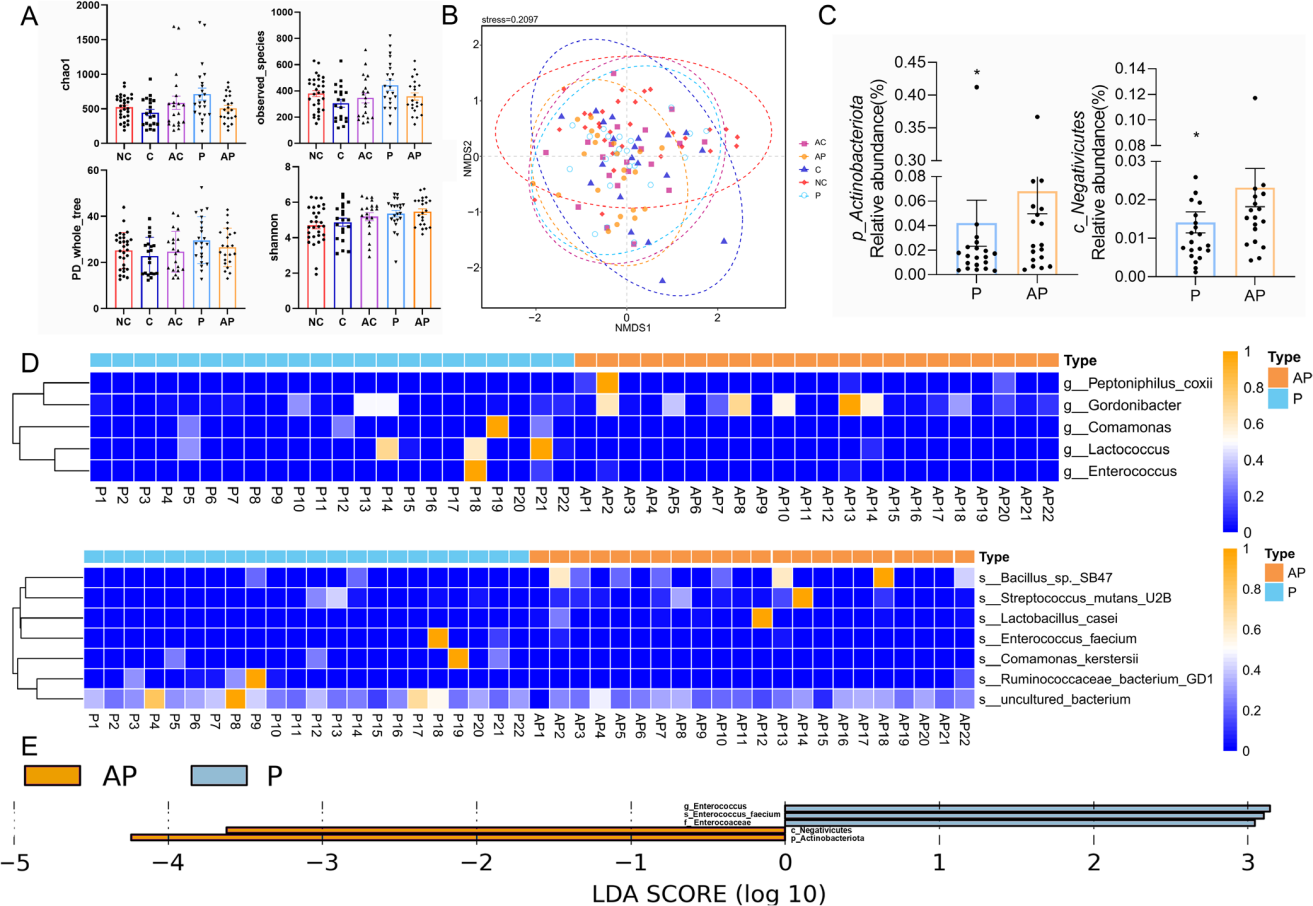
Changes in gut microbiota after probiotics treatment. (A) Alpha diversity indices (Chao 1, observed_species, PD_whole_tree, and Shannon) show diversity and evenness of gut microbiota. (B) Beta diversity analysis (between‐sample microbial community differences) visualized via Non‐metric Multidimensional Scaling (NMDS). (C) The microorganisms with significant differences between group P and group AP at the phylum and class levels. (D) Heat maps show differences at the genus and species levels. (E) LEfSe revealed statistically significant shifts in gut microbiota following probiotic intervention. AP, After intervention of probiotic group; AC, After observation of control group; C, Control group; NC, Normal control group; P, Probiotic group. *p < 0.05.

To evaluate probiotics effects on the microbiota community abundance, alpha diversities were analyzed as shown in Figure 2A [22]. No significant change occurred in alpha diversity indices (Chao 1, observed‐species, Shannon, or PD‐whole‐tree) after probiotics consumption. Beta diversity was further assessed with NMDS (Figure 2B). ANOSIM showed no statistical difference between the probiotics (P) and after probiotics treatment (AP) groups (*R* = −0.02, *p* = 0.759), indicating that probiotics do not cause drastic changes in the gut microbiota of PD patients. The *p_Actinobacteriota* and *c_Negativicutes* abundance increased significantly after probiotics treatment at the phylum and class level (Figure 2C). At the genus level, the abundance of *Gordonibacter* and *Peptoniphilus coxii* was significantly higher after probiotics treatment, while the abundance of *Lactococcus*, *Comamonas*, *and Enterococcus* significantly decreased (Figure 2D). At the species level, the abundance of *Bacillus* sp._SB47, Streptococcus_mutans_U2B, and *Lactobacillus_casei* increased, while the abundance of *Enterococcus*_*faecium*, *Comamonas_kerstersii*, and *Ruminococcaceae_bacterium GD‐1* decreased (all *p* < 0.05; Figure 2D). The LDA identified *Actinobacteria* and *Negativicutes* as post‐intervention biomarker bacteria in PD patients (Figure 2E).

**FIGURE 3:**
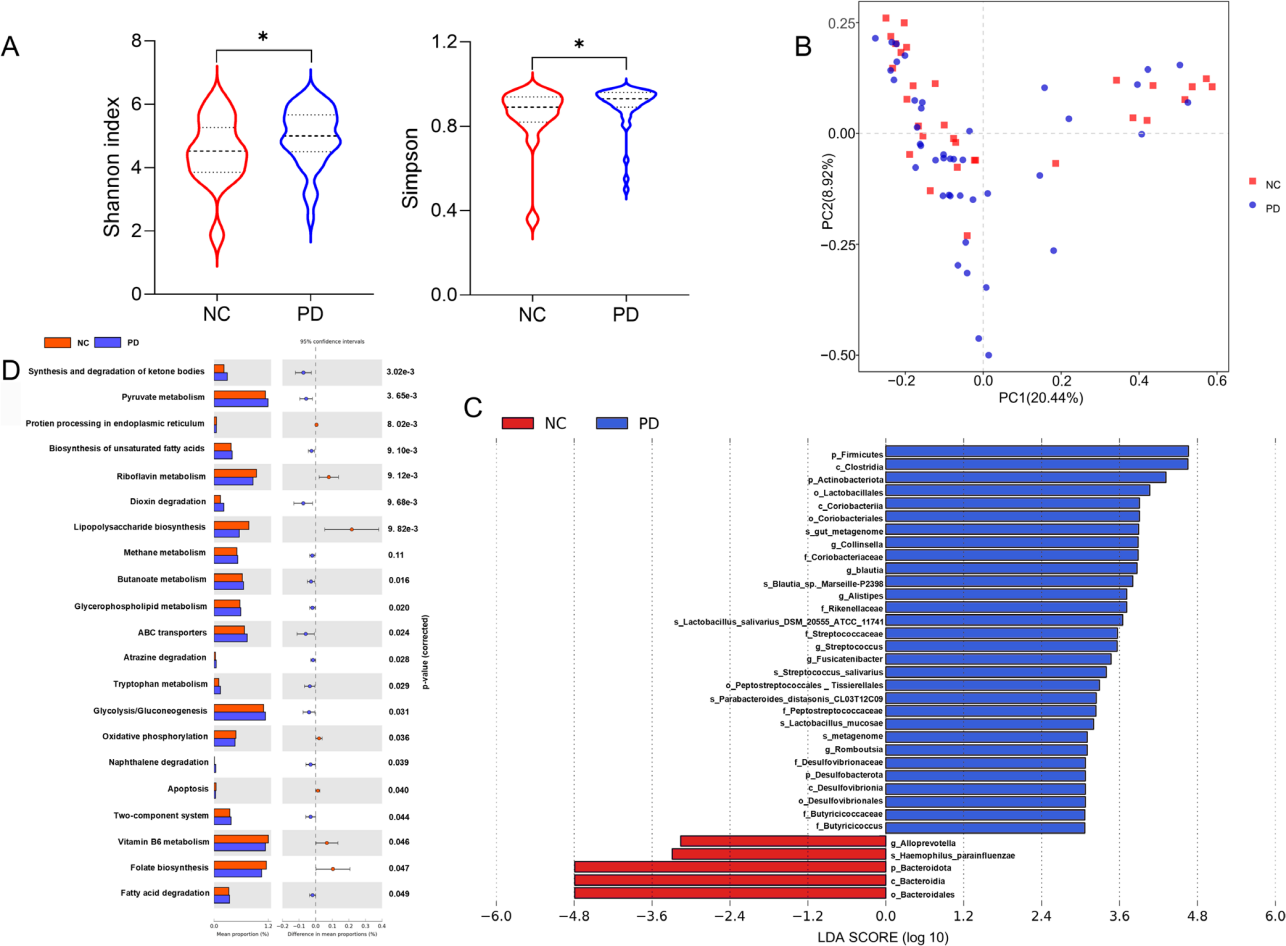
Alteration of gut microbiota in PD. (A) Alpha diversity indices (Shannon and Simpson) show changes in the diversity of the gut microbiota in PD patients compared to normal controls. (B) Beta diversity analysis (between‐sample microbial community differences) visualized via Principal component analysis (PCoA). (C) LEfSe analysis identified the microbes whose abundances significantly differed between the PD and NC groups. (D) 21 KEGG level 3 pathways significantly differed between the PD and NC groups. NC, Normal control group; PD, PD group. *p < 0.05.

Meanwhile, we conducted a comparison of the gut microbiota differences between PD patients (*n* = 42) and normal controls (*n* = 30). Shannon (*p* = 0.038) and Simpson indices (*p* = 0.017) show significantly higher microbial diversity in PD patients (Figure 3A). Beta diversity was further assessed with PCoA (Figure 3B). ANOSIM analysis showed marginally greater intergroup (PD vs. control) than intragroup variation, but the difference was not statistically significant (*R* = 0.04, *p* = 0.084). We used LEfSe to identify bacteria showing significant differential abundance between groups (Figure 3C). *g_Alloprevotella*, *s_Haemophilus_parainfluenzae*, and *Bacteroidota* were identified as biomarkers in the NC group, whereas *p_Firmicutes*, *c_Clostridia*, and *p_Actinobacteriota* among 30 taxa were identified as biomarkers in the PD group. We further utilized PICRUSt 2 to predict microbiota functions, identifying 21 KEGG pathways that differed significantly between the two groups (level 3; Figure 3D). The PD group exhibited significant enrichment in 15 metabolic pathways, including Synthesis and degradation of ketone bodies, Biosynthesis of unsaturated fatty acids, Dioxin degradation, Pyruvate metabolism, Butanoate metabolism, Glycolysis/Gluconeogenesis, and Tryptophan metabolism in comparison to the control group.

### 3.4 Serum Metabolites Change in PD

**FIGURE 4:**
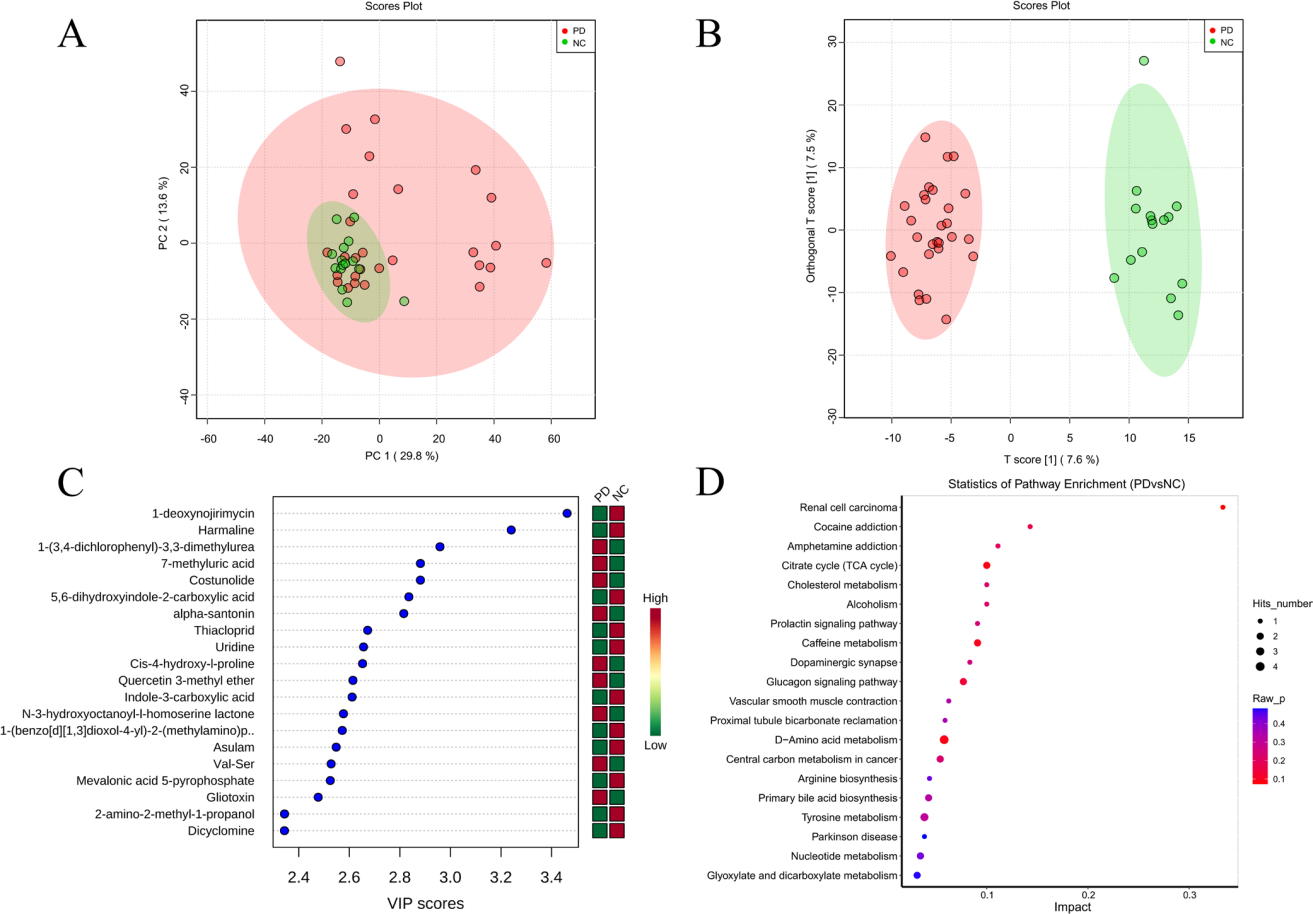
Metabolomic analysis in PD patients and normal controls, and the analysis of metabolic pathways. (A) PCA analysis describes trends in the overall distribution between samples in the PD and NC groups. (B) OPLS‐DA revealed significant intergroup discrimination between PD and NC groups. (C) VIP value plots of the differential metabolites (top 20 based on VIP value). (D) The KEGG differential metabolic pathway plot (top 20 enriched metabolic pathways based on hit count). PD, PD group; NC, Normal control group.

A total of 28 PD patients (probiotics group: *n* = 15; control group: *n* = 13) and 15 normal control people provided serum samples. PD patients who provided serum samples showed no significant differences in general characteristics, PD severity, or LEDD compared to all 50 PD patients (Table S1). We previously showed that PD patients exhibit a different gut microbiota composition compared to normal controls. To gain deeper insights, we investigated the variations in serum metabolites between them. PCA and OPLS‐DA can observe differences in metabolites between the two groups (Figure 4A,B). To identify differentially metabolites between groups, we screened for metabolites with a combination of VIP values > 1 and *p*‐values < 0.05 using OPLS‐DA analysis. Figure 4C shows the top 20 differential metabolites ranked by VIP scores. Compared to the normal control group, 9 differential metabolites, such as 7‐methyluric acid, Costunolide, and alpha‐santonin in the PD group were significantly increased, while 11 differential metabolites, such as 1‐Deoxynojirimycin, Harmaline, Dicyclomine, Asulam, Uridine, and Thiacloprid were significantly decreased (Figure 4C). Notably, theophylline levels were significantly lower in PD patients despite not ranking among the top 20 metabolites. To elucidate the biological functions of serum differential metabolites, we conducted metabolic pathway enrichment analysis. The top 20 significantly altered metabolic pathways are displayed in Figure 4D.

### 3.5 Probiotics Intervention Alters Serum Metabolites in PD Patients

**FIGURE 5:**
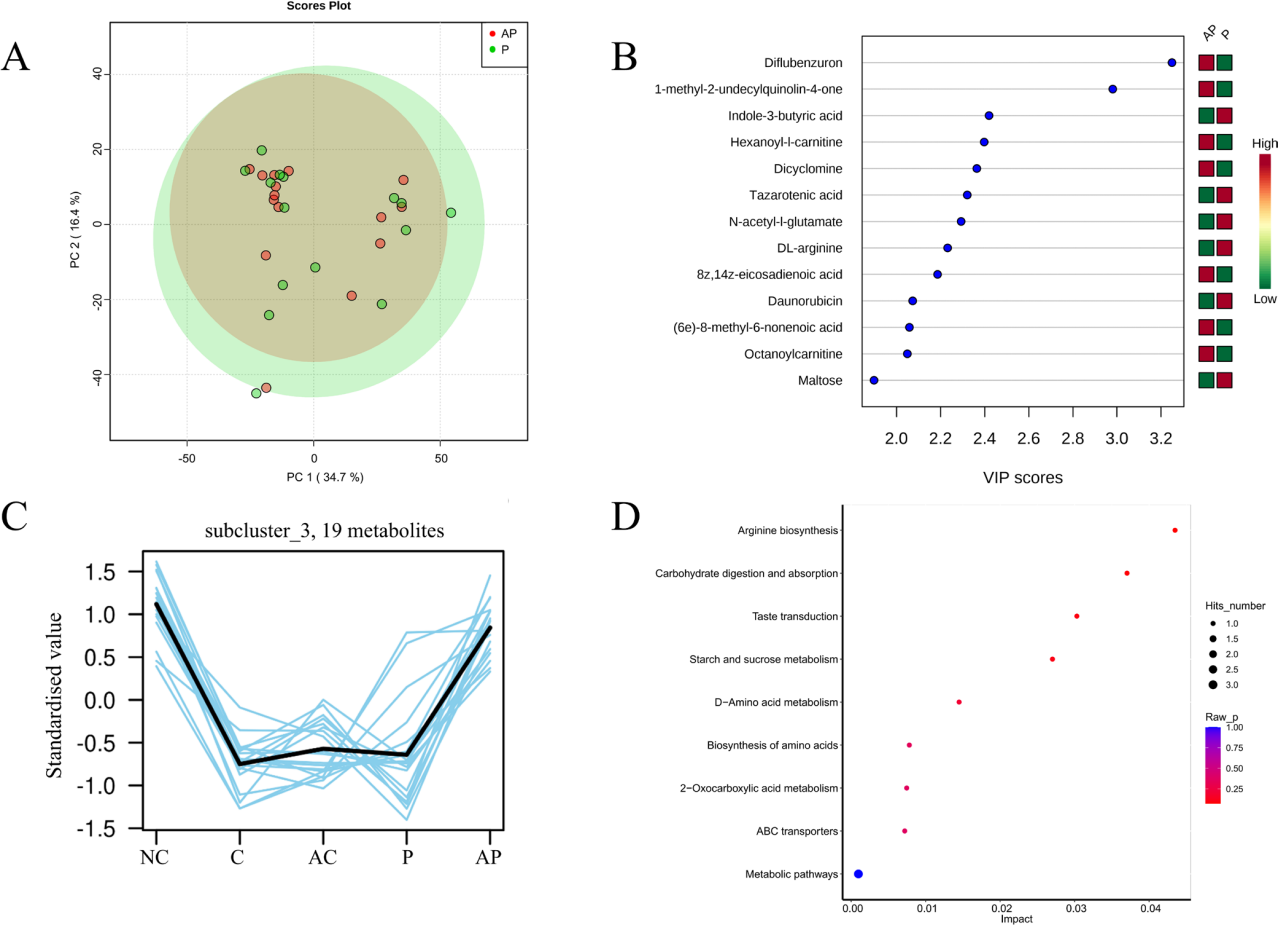
Metabolites and metabolic pathways analysis in the probiotics group. (A) PCA analysis of serum metabolites, depicting clustering patterns between pre‐ and post‐intervention groups. (B) The VIP value plots of the differential metabolites. (C) K‐means subcluster analysis was performed to assess trends in the relative abundance of metabolites across experimental groups. (D) KEGG pathway enrichment analysis, highlighting significantly perturbed metabolic pathways. AP, After intervention of probiotic group; AC, After observation of control group; C, Control group; NC, Normal control group; P, Probiotic group.

After probiotics intervention, some intestinal bacteria in PD patients showed significant changes. Microbial metabolites may act as primary mediators in regulating the microbiota‐gut‐brain axis: after intestinal absorption, these metabolites enter systemic circulation and ultimately reach the brain to exert biological effects. To test this hypothesis, serum metabolites in the probiotics treatment and control groups were sequenced by untargeted metabolomics. PCA analysis showed that the serum metabolites of PD patients were different after probiotics intervention (Figure 5A). We found that up‐regulated metabolites after probiotics intervention were Octanoylcarnitine, 8Z,14Z‐eicosadienoic acid, Diflubenzuron, (6e)‐8‐methyl‐6‐nonenoic acid, while down‐regulated metabolites included Tazarotenic acid, DL‐arginine, N‐acetyl‐l‐glutamate, Daunorubicin, Indole‐3‐butyric acid, and Maltose (Figure 5B). K‐means cluster analysis showed that the levels of 19 metabolites (including Theophylline, Urushiol I, and Lauroyl‐l‐carnitine) exhibited a directional shift toward those observed in normal controls following probiotics administration (Figure 5C, Table S2). KEGG enrichment analysis of differential metabolites highlighted nine significantly altered pathways (*p* < 0.05): Arginine biosynthesis, Carbohydrate digestion and absorption, Taste transduction, Starch and sucrose metabolism, D‐Amino acid metabolism, Biosynthesis of amino acids, 2‐Oxocarboxylic acid metabolism, ABC transporters, and Metabolic pathways (Figure 5D).

### 3.6 Association Between Clinical Scores, Intestinal Flora, and Serum Metabolites in PD Patients

**FIGURE 6:**
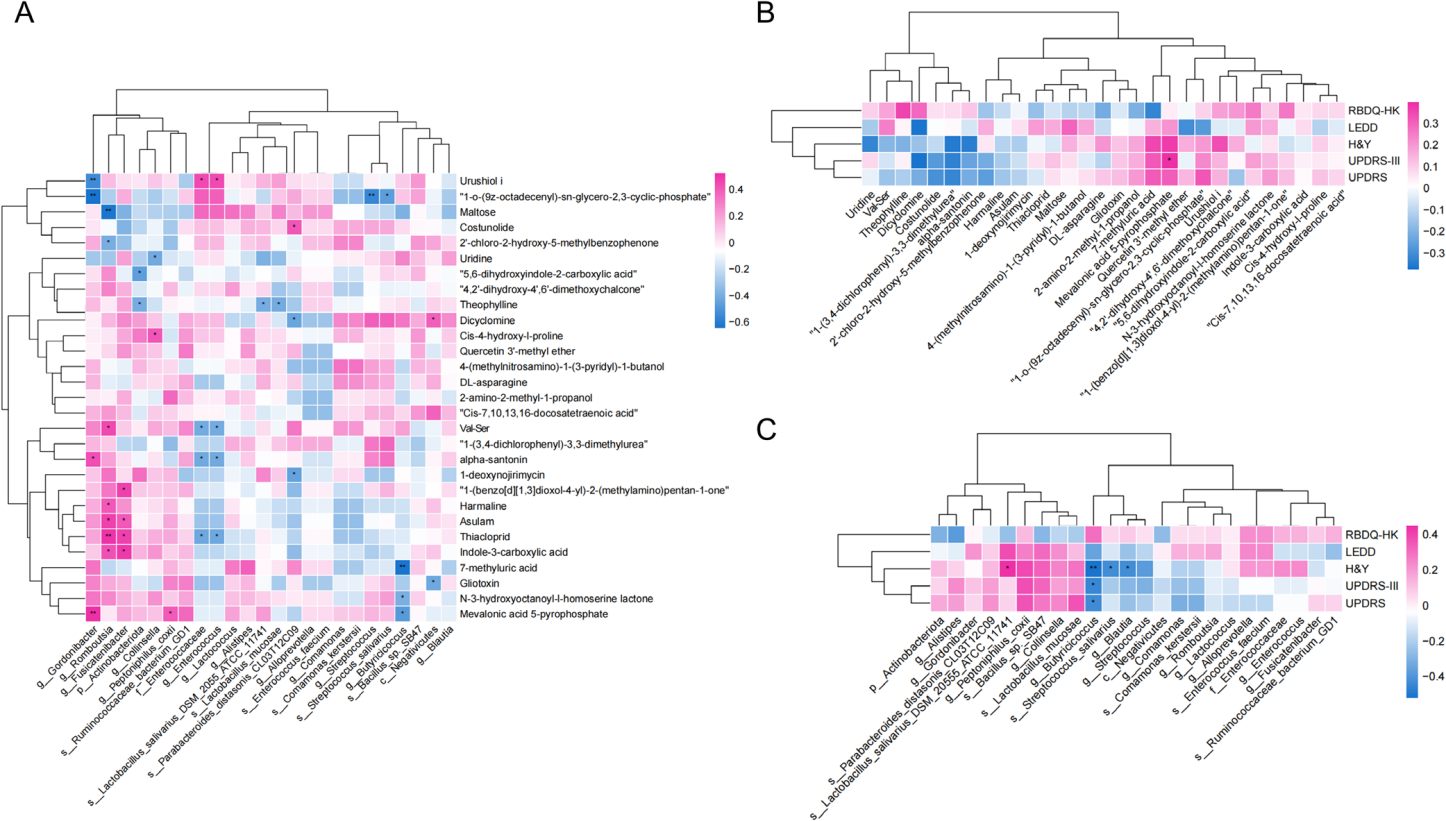
Correlations among clinical scores, gut microbiota, and plasma metabolites in PD patients. (A) Association between plasma metabolites and gut microbiota. (B) Association between plasma metabolites and clinical scores. (C) Association between clinical scores and gut microbiota. *p < 0.05. **p < 0.01.

Multivariate correlation analyses were conducted to explore the relationship between gut flora and serum metabolites, which were significantly different after probiotics intervention, and clinical measures (UPDRS, UPDRS‐III, RBD‐HK, LEDD, H‐Y) in PD patients. Our results showed that *Romboutsia* and *Fusicatenibacter* exhibited positive correlations with Asulam, Thiacloprid, and others. In contrast, *Butyricoccus* was negatively correlated with 7‐methyluronic acid, N‐3‐hydroxyoctanoyl‐l‐homoserine lactone, and Mevalonic acid 5‐pyrophosphate (Figure 6A). Mevalonic acid 5‐pyrophosphate was positively correlated with UPDRS (Figure 6B). *Butyricoccus* was negatively correlated with UPDRS, UPDRS‐III, and H‐Y. Additionally, *Streptococcus salivarius* and *Blautia* were also found to be negatively correlated with H‐Y (Figure 6C).

## 4 Discussion

A hallmark pathological feature of PD is the misfolding and abnormal aggregation of α‐syn, which results in the demise of dopaminergic neurons. Braak first hypothesized that α‐syn could be transmitted to the midbrain via the gastrointestinal vagus nerve [27, 28]. Supporting this, Kim et al. [29] injected pathological α‐syn into the gastrointestinal muscles of healthy mice. Seven months later, the injected α‐syn was detected in the striatum, and the mice showed dopamine system impairment. In 2014, the first study analyzing gut microbiota composition via 16S rRNA sequencing in 72 PD patients and matched healthy controls revealed significant microbial community differences between groups [30]. These findings collectively indicate that gut microbiota may play a critical role in PD pathogenesis.

In this trial, PD patients received a probiotic formulation containing four strains: *Bacillus licheniformis*, *Bifidobacterium longum* , *Lactobacillus acidophilus* , and *Enterococcus faecalis* . After 12 weeks of probiotics treatment, the UPDRS scores decreased in PD patients, and LEDD was reduced in the probiotics group. Another study [31] demonstrated that probiotics improved MDS‐UPDRS scores in PD patients. However, variations in probiotic strains and dosing regimens between studies limit the comparability of these results. In addition, in this study, we found that probiotics supplementation improved RBD symptoms and reduced RBDQ‐HK scores in PD patients. The probiotics used in this trial had a good safety profile. Some participants voluntarily continued taking the probiotics after the trial concluded and tolerated them well.

Increasing numbers of preclinical and clinical studies indicate that supplementing probiotics can ameliorate motor symptoms, constipation, anxiety, and depression in PD patients [18, 32]. To our knowledge, this study is the first to provide clinical evidence demonstrating that probiotics can improve RBD symptoms in PD patients. RBD is not only a typical non‐motor symptom of PD but also an important biomarker during its prodromal phase [33]. Recent evidence suggests that PD may have two distinct origins based on its pathological changes: one originating from the central nervous system and the other from the peripheral autonomic nervous system. These origins are categorized as “brain‐first” and “body‐first” pathways, respectively [34]. Research indicates that PD‐RBD patients predominantly present as the “body‐first” subtype, exhibiting initial abnormalities in the enteric parasympathetic nervous system function before the onset of dopamine reduction in the brain [35]. Moreover, emerging evidence indicates that gut microbiota alterations in RBD patients mirror those observed in early‐stage PD; decreased abundance of *Butyricicoccus* and *Faecalibacterium* may represent potential markers for the progression from RBD to PD [36]. Therefore, gut microbiota disturbance may predispose patients to develop RBD and subsequently progress to PD. Probiotics intervention shows potential for ameliorating RBD symptoms through gut microbiota modulation. In addition, whether probiotics can slow the neurodegeneration process of iRBD deserves further study.

More importantly, we further investigated the mechanisms by which probiotics improve clinical symptoms in PD patients through analysis of gut microbiota and plasma metabolism. First, fecal analysis revealed that the intestinal flora of PD patients differed from normal controls, with increased microbial α‐diversity observed in the PD cohort. Multiple studies with different sample sizes and different populations have consistently demonstrated that PD patients exhibit dysbiosis of gut flora, but the specific bacterial taxa showing differential abundance vary between studies [37, 38, 39, 40]. Several studies have shown a reduction in *Prevotella* and an increase in *Bifidobacterium* in PD. In this experiment, we also found decreased *Alloprevotella* in the PD group, but no significant increase in *Bifidobacterium*. The variability observed across studies may be attributed to several factors, including differences in sample size, dietary patterns, geographic location, and race. The α‐diversity and β‐diversity of gut microbiota showed no significant changes following probiotic intervention, which is consistent with prior research findings. These findings suggest that supplementing probiotics does not cause substantial perturbations in overall gut microbiota composition among PD patients, but rather exerts its therapeutic effects through altering specific functional bacteria. The abundance of *Actinobacteria*, *Negativicutes*, *Peptoniphilus_coxii*, *Gordonibacter*, *Bacillus_sp._SB47*, *Streptococcus_mutans_U2B*, and *Lactobacillus_casei* increased, while the abundance of *Lactococcus*, *Enterococcus*_*faecium*, *Comamonas_kerstersii*, and *Ruminococcaceae_bacterium GD‐1* decreased in fecal specimens from PD patients after probiotics supplementation. No notable difference was observed in the vast majority of bacteria, showing the inherent resilience of intestinal microorganisms in PD patients.

Correlation analyses revealed significant negative associations between *Butyricoccus* abundance and clinical measures, including UPDRS, UPDRS‐III, and H‐Y. *Butyricoccus is* a common probiotic that works by producing short‐chain fatty acids such as butyric acid [41]. In the MPTP‐induced PD mouse model, *Butyricoccus* improved motor function and reduced the loss of dopaminergic neurons [42]. Engineering strain *Clostridium butyricum‐GLP‐1* enhanced motor function by upregulating TH expression and downregulating α‐syn expression and restoring gut microbiota balance [43]. Also, decreased abundance of *Butyricoccus* may represent potential markers for the progression from RBD to PD. These findings position *Butyricoccus* as a promising probiotic candidate for PD therapeutics, with mechanisms potentially involving dopaminergic protection and microbiota‐gut‐brain axis modulation.

Metabolomics analysis in this study found reduced serum theophylline levels in the PD group compared to normal controls, which is consistent with prior findings by Ohmichi et al. [44]. Theophylline acts as a non‐selective receptor antagonist of adenosine. Adenosine A2A receptors can form a heterodimer with the dopamine D2 receptor, and activation of the A2A receptor can inhibit the effect of dopamine [45]. Previous research has demonstrated that the A2A receptor antagonist could improve clinical symptoms in PD patients and reduce daily OFF time [46, 47, 48]. In this study, K‐means cluster analysis revealed that theophylline levels were restored after probiotics intervention. As an adenosine receptor antagonist, theophylline may activate dopamine D2 receptors via A2A receptor inhibition, suggesting that probiotics may exert part of their therapeutic effect by elevating theophylline levels. Arginine is a precursor for nitric oxide (NO), and excessive NO production can lead to oxidative stress and mitochondrial dysfunction, both of which play key roles in the pathogenesis of PD [49]. Previous studies have found that exercise can have a therapeutic effect on PD by inhibiting arginine biosynthesis and NO production [50, 51]. This study also found that serum L‐arginine levels in PD patients were significantly reduced after probiotic treatment. KEGG analysis further revealed that the arginine biosynthesis pathway was significantly affected, suggesting that probiotic supplementation improved amino acid metabolism in PD patients. In addition, PD is associated with glucose metabolism disorders [52]. This study demonstrated that probiotic treatment could modulate these disturbances by affecting pathways related to carbohydrate digestion and absorption, starch and sucrose metabolism, and 2‐hydroxycarboxylic acid metabolism. Together, these results indicate that probiotic supplementation improved both amino acid metabolism and energy metabolism in PD patients.

The human gut microbiota likely influences levodopa efficacy. It was reported that *Enterococcus faecalis* can decarboxylate levodopa to dopamine in the intestine, thereby reducing the amount of dopamine that ultimately reaches the central nervous system [53]. Although the probiotics used in this study contained *Enterococcus faecalis* , LEDD decreased, and the symptoms of PD patients improved following 12 weeks of probiotics treatment. Notably, microbiome analysis revealed no corresponding increase in *Enterococcus faecalis* abundance, suggesting these effects may result from complex interactions of intestinal bacteria. The mechanism underlying the LEDD reduction is unclear. Is it due to probiotics influencing levodopa metabolism, or through the microbiome‐gut‐brain axis? Further levodopa pharmacokinetic studies could be performed to clarify whether multi‐strain probiotics affect levodopa metabolism.

There are some limitations in this study. First, the intervention period was limited to 12 weeks, and the sample size was small; therefore, larger and longer‐term follow‐up studies are needed to confirm the findings. Second, microbial composition varies across intestinal regions, and fecal microbiota only represents the distal gut community rather than the entire gut microbiota. Third, the effect of probiotics on levodopa pharmacokinetics remains unclear, and further research is needed.

## 5 Conclusion

In summary, our research demonstrated that PD patients exhibit gut microbiota dysbiosis; 12‐week probiotics supplementation in PD patients improved both motor symptoms and RBD symptoms. Furthermore, the probiotics intervention was found to partially modulate gut microbial composition and plasma metabolite profiles.

## Author Contributions

**Yitong Du:** design and execution of data analysis; preparation of the initial manuscript draft. Houzhen Tuo: project administration; manuscript review. **Lin Wang, Ying Cui, Xiaojiao Xu, Mingkai Zhang, Yue Li, Ting Gao, Dan Gao, Zhi Sheng, and Shiya Wang:** conduct of the research project and data collection. All authors contributed to the preparation of the manuscript and approved the final version for submission.

## Conflicts of Interest

All authors declare no conflicts of interest.

## Supporting information


**Table S1:** Clinical characteristics of PD patients who provided serum samples.

**Table S2:** Kmeans‐subcluster.

## Data Availability

Datasets are available from the corresponding author upon reasonable request.
