# Supplementary material for: Effect of Probiotics Supplementation on REM Sleep Behavior Disorder and Motor Symptoms in Parkinson's Disease: A Pilot Study
Source: CNS Neurosci Ther. 2025 Jul 27;31(7):e70541. doi: 10.1111/cns.70541 (PMC12301574; doi:10.1111/cns.70541)
Supplement: Supplementary file 1 — Table S1: Clinical characteristics of PD patients who provided serum samples. [file CNS-31-e70541-s002.docx]

| Table S1. Clinical characteristics of PD patients who provided serum samples. | | | |
| --- | --- | --- | --- |
| Characteristics | Serum subpopulation  (n=28) | All PD patients  (n=50) | *P*-value |
| Age (years) | 65.5 (61.3, 71) | 65.5 (61, 73.3) | 0.83^a^ |
| Gender (% male) | 53.6 | 60 | 0.58^b^ |
| H&Y | 2 (1.5, 2.5) | 2 (1.9, 2.5) | 0.41 ^a^ |
| UPDRS-Ⅲ | 15.3 (10.5, 25.8) | 16.3 (12.4, 25.3) | 0.67 ^a^ |
| UPDRS | 25 (18, 38) | 30 (21, 38.3) | 0.48 ^a^ |
| RBD-HK | 33.7 ± 13.7 (n=18) | 32 (22.5, 43.5) (n=38) | 0.9 ^a^ |
| LEDD (mg/day) | 337.5 (150, 450) | 337.5 (168.8, 450) | 0.8 ^a^ |

^a^ P-values obtained from Mann-Whitney U test. ^b^ P-values obtained from Chi-square test.
