# Supplementary material for: Effect of Probiotics Supplementation on REM Sleep Behavior Disorder and Motor Symptoms in Parkinson's Disease: A Pilot Study
Source: CNS Neurosci Ther. 2025 Jul 27;31(7):e70541. doi: 10.1111/cns.70541 (PMC12301574; doi:10.1111/cns.70541)
Supplement: Supplementary file 2 — Table S2: Kmeans‐subcluster. [file CNS-31-e70541-s001.docx]

| Table S2. Kmeans-subcluster | | | | | |
| --- | --- | --- | --- | --- | --- |
| Metabolites | NC | C | AC | P | AP |
| Isopropalin | 1.309 | -1.201 | 0.001 | -0.702 | 0.593 |
| 1-methyl-2-undecylquinolin-4-one | 1.000 | 0.0888 | -0.428 | -1.402 | 0.918 |
| Decanoyl-l-carnitine | 1.193 | -0.727 | 0.177 | -1.138 | 0.849 |
| (6e)-8-methyl-6-nonenoic acid | 0.456 | -0.354 | -0.363 | -1.192 | 1.454 |
| Theophylline | 1.138 | -1.107 | 0.939 | 0.149 | 0.759 |
| 2-(3-hydroxyoctyl)-5-oxo-1-pyrrolidineheptanoic acid | 1.506 | -0.624 | -0.603 | 0.824 | 0.546 |
| Cis-7,10,13,16-docosatetraenoic acid | 0.903 | 0.796 | -1.034 | -0.262 | 1.189 |
| Dicyclomine | 1.303 | -0.691 | -0.738 | -0.735 | 0.861 |
| Urushiol i | 0.393 | -1.269 | -0.835 | 0.661 | 1.050 |
| 1-o-(9z-octadecenyl)-sn-glycero-2,3-cyclic-phosphate | 0.564 | -1.268 | -0.893 | 0.788 | 0.810 |
| Octanoylcarnitine | 1.248 | -0.598 | 0.059 | -1.272 | 0.681 |
| N-achidonylmaleimide | 1.052 | -0.809 | 0.219 | -1.058 | 1.035 |
| Hexanoyl-l-carnitine | 1.106 | -0.559 | 0.281 | -1.217 | 0.951 |
| Napelline | 1.244 | -0.694 | -0.835 | -0.642 | 0.928 |
| Lauroyl-l-carnitine | 1.521 | -0.873 | 0.321 | -0.783 | 0.456 |
| Trihexyphenidyl | 1.616 | -0.572 | 0.632 | 0.743 | 0.331 |
| Leukotriene b4 dimethylamide | 1.147 | -0.708 | -0.765 | -0.715 | 1.042 |
| 8z,14z-eicosadienoic acid | 0.975 | -0.762 | 0.812 | -0.603 | 1.202 |
| 6-[[2-[[4-(2,4-dichlorophenyl)-5-(5-methyl-1h-imidazol-2-yl)-2-pyrimidinyl]amino]ethyl]amino]-3-pyridinecarbonitrile | 1.578 | -0.545 | -0.913 | 0.491 | 0.371 |
